# Micro-Mechanical Response of an Al-Mg Hybrid System Synthesized by High-Pressure Torsion

**DOI:** 10.3390/ma10060596

**Published:** 2017-05-30

**Authors:** Megumi Kawasaki, Jae-il Jang

**Affiliations:** 1 Division of Materials Science and Engineering, Hanyang University, Seoul 04763, Korea; megumi@hanyang.ac.kr; 2 Departments of Aerospace & Mechanical Engineering and Materials Science, University of Southern California, Los Angeles, CA 90089-1453, USA; mkawasak@usc.edu

**Keywords:** intermetallic composite, grain refinement, hardness, high-pressure torsion, nanocomposite, nanoindentation, plasticity

## Abstract

This paper summarizes recent efforts to evaluate the potential for the formation of a metal matrix nanocomposite (MMNC) by processing two commercial bulk metals of aluminum and magnesium alloy through high-pressure torsion (HPT) at room temperature. After significant evolutions in microstructures, successful fabrication of an Al-Mg hybrid system was demonstrated by observing unique microstructures consisting of a multi-layered structure and MMNC. Moreover, the evolution of small-scale mechanical properties was examined through the novel technique of nanoindentation and the improvement in plasticity was estimated by calculating the strain rate sensitivity of the Al-Mg hybrid system after HPT. The present paper demonstrates that, in addition to conventional tensile testing, the nanoindentation technique is exceptionally promising for ultrafine-grained materials processed by HPT, where the samples may have small overall dimensions and include heterogeneity in the microstructure.

## 1. Introduction

The processing of metals through the application of severe plastic deformation (SPD) provides the potential for achieving exceptional grain refinement in bulk solids. It is now well accepted that ultrafine-grained (UFG) materials are defined as having submicron grains in the range of 100–1000 nm or nanocrystalline grains with sizes in the range of 10–100 nm [1]. Among the reported SPD techniques, one of the most attractive methods refers to the processing by high-pressure torsion (HPT) [2], where this type of processing leads to exceptional grain refinement that is not generally achieved using other procedures [3]. Therefore, HPT has been applied for the consolidation of metallic powders [4,5,6,7,8] and bonding of machining chips [9,10], whereas very limited reports examined the application of HPT for the fabrication of hybrid systems including nanocomposites [11,12].

The fundamental principles of HPT processing were described in detail in an earlier review [2]. Specifically, the sample in a disk shape is applied to receive both very high compressive straining and torsion straining concurrently. Numerous reports are now available demonstrating that bulk materials after HPT having ultrafine and nanometer grain sizes generally show superior mechanical properties, which include high strength dictated through the Hall–Petch relationship at low temperatures and a superplastic forming capability at high temperatures due to possible fast diffusion.

The development of micro-mechanical behavior is observed after significant changes in microstructure through SPD processing and it is of great importance for obtaining practical future applications of these UFG metals. Accordingly, recent developments in characterization techniques lead to a better understanding of the evolution in the mechanical properties of UFG materials processed by SPD. In particular, the novel technique of nanoindentation has become a common tool for the simultaneous measurement of a number of mechanical properties by using a small microstructural scale [13]. Thus, there have been several studies reporting the use of the nanoindentation technique for investigating the plastic deformation properties of SPD-processed metals including Al alloys [14,15,16,17,18], pure Cu [19], pure Cr [20], a ZK60 magnesium alloy [21], pure Nb [22], a Pb-62%Sn eutectic alloy [23], a Zn-22% Al eutectoid alloy [24] and high entropy alloys (HEAs) [25,26,27], while numerous studies demonstrated conventional mechanical testing in tension and compression. A recent review summarized the available experimental results showing the enhancement in the micro-mechanical response at room temperature (RT) observed by the nanoindentation technique in a range of metals and alloys after several different SPD processing procedures [28].

Accordingly, a new approach of applying conventional HPT processing was demonstrated under quasi-constrained conditions [29,30] for synthesizing a hybrid system and ultimately forming an intermetallic-based metal matrix nanocomposite (MMNC) from separate conventional metals of Al and Mg through diffusion bonding at RT by HPT. It should be emphasized that the synthesis of intermetallic compounds in the present study involves the bulk-state reaction of metals [31], which is different from the procedure of mechanical alloying of powder through the consolidation of powders using HPT [32,33]. The unique microstructure and exceptional hardness was demonstrated in the Al-Mg hybrid system synthesized by HPT through 20 turns. A study of post-deformation annealing (PDA) was applied to determine the microstructural change and influence in mechanical properties in the synthesized alloy system. Moreover, the enhancement in micro-scale deformation behavior was examined through the nanoindentation experiments on the Al-Mg system after HPT and after PDA. Special emphasis is placed on demonstrating the evolution of the micro-mechanical responses in the hybrid system by measuring the strain rate sensitivity.

## 2. Synthesis of an Al-Mg Hybrid Metal System through Diffusion Bonding

### 2.1. Microstructural Evolution and Hardness Development

**Figure 1:**
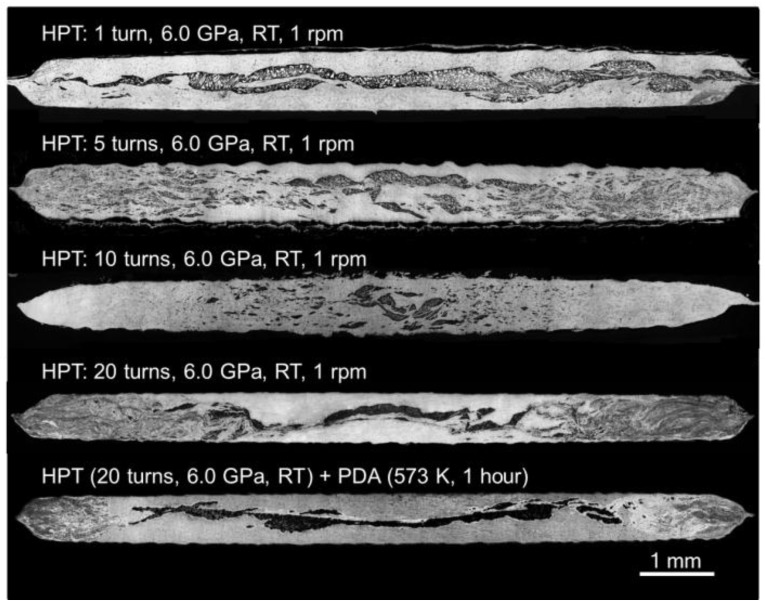
The vertical cross-sections of the Al–Mg system after HPT under a pressure of 6.0 GPa at room temperature for, from the top, 1, 5, 10, 20 turns and for 20 HPT turns followed by PDA [34,35].

Figure 1 shows optical micrographs demonstrating overviews of the microstructure taken at the cross-sections of the Al-Mg disks after HPT for 1, 5, 10 and 20 turns and 20 HPT turns followed by PDA at 573 K for 1 h from the top [34,35].

A disk after one turn showed a multi-layered structure with fragmented Mg layers with thicknesses of ~200 µm without any segregation at the Al-Mg interfaces throughout the disk diameter. A similar formation of multi-layered microstructure was observed at the central regions at *r* < 2.0 mm after five turns and ~1.0 mm after 10 and 20 turns, where *r* denotes the radius of the HPT disk. By contrast, the disk peripheries at *r* > 2.5 mm after five turns demonstrated a homogeneous distribution of very fine Mg phases having thicknesses of ~5–10 µm to even true nano-scale sizes of ~100–500 nm within the Al matrix. Furthermore, these fine Mg phases disappeared at the disk edges, and there was no evidence of visible Mg phases at ~3< *r* < 5 mm after 10 and 20 turns. A similar microstructure of multi-layered formation towards complete mixture of Al-Mg phases along the radial direction was observed after HPT for 20 turns followed by PDA, whereas the outer region was reduced. Accordingly, the synthesized Al-Mg system after HPT and after PDA consists of gradient-type microstructures involving microstructural heterogeneity across the disk diameters.

**Figure 2:**
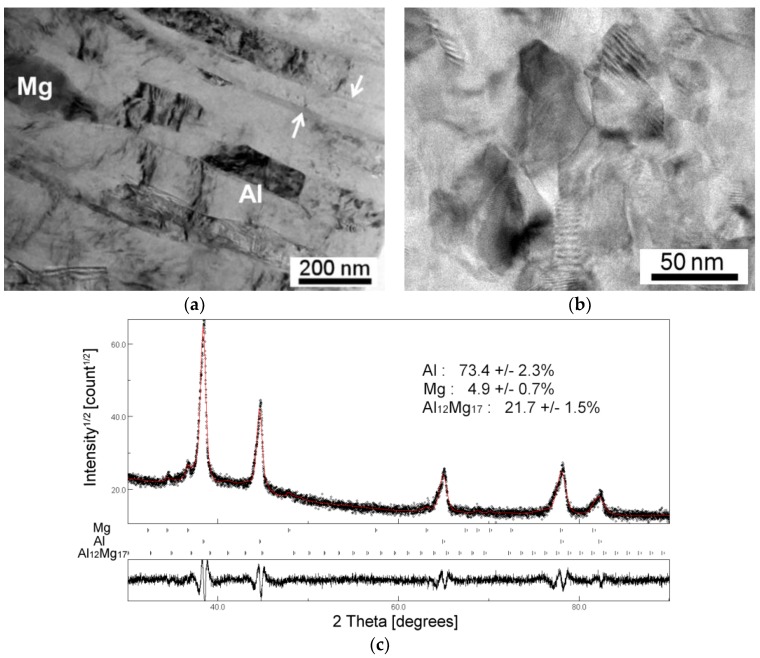
TEM bright-field images taken at the Al-Mg disk edges after HPT for (**a**) five turns and (**b**) 10 turns and (**c**) the XRD profile with the MAUD estimation for the disk edge after 10 HPT turns [34].

**Figure 3:**
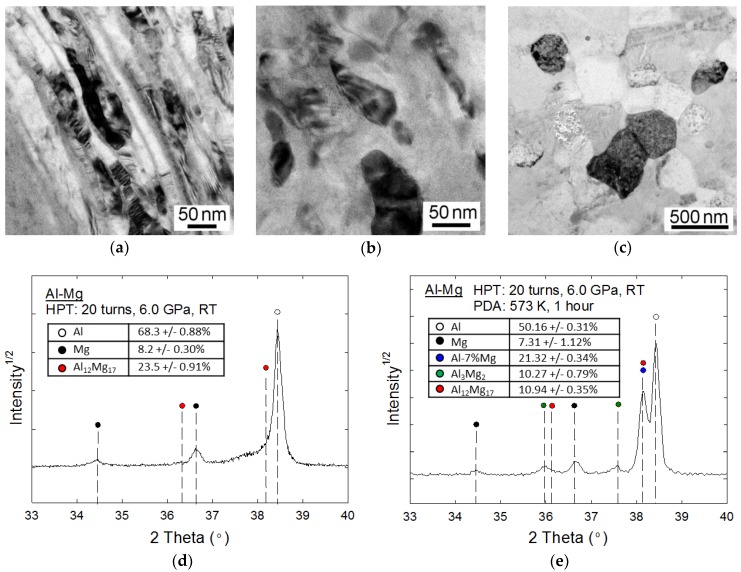
Representative TEM bright-field images taken at the disk edges after (**a**,**b**) HPT for 20 turns and (**c**) HPT followed by PDA in the Al-Mg system and the X-ray diffraction profiles for the disk edges of the Al-Mg system after (**d**) HPT for 20 turns and (**e**) HPT and PDA [37].

Detailed microstructural analysis was conducted at the disk edges of *r* ≈ 4.0–4.5 mm and the results are shown in Figure 2 and Figure 3 for the Al-Mg system after HPT for 5–10 turns and 20 turns and additional PDA, respectively. After five turns of HPT, true nanostructure was already achieved as shown in Figure 2a, where a layered microstructure is demonstrated at the edge of the Al-Mg disk. The layers have thicknesses of 90–120 nm, and these layers contain numerous dislocations subdividing the layers in a vertical sense. The measurements showed an average grain size of ~190 nm in the Al matrix phase. In this micrograph, a single Mg phase is visible that has a rigid bonding interface with the Al matrix without any visible voids. Moreover, within the Al matrix phase, several thin nano-layers are observed with an average thickness of ~20 nm as indicated by the white arrows.

Figure 2b shows a finer microstructure after 10 HPT turns at the edge of the Al-Mg disk. There was no evidence of an Mg-rich phase and an average grain size of ~90 nm was observed where, although it is not included in the micrograph, there was a similar type of thin layers as shown in the disk edge after five turns. An earlier study examined these thin layers in the Al matrix closely by element mapping and quantitative chemical analysis [34]. The detailed analysis revealed that the thin layers are composed of an intermetallic compound of β-Al_3_Mg_2_ that has a low density of ~2.25 g cm^−3^. Since the thin layers existed randomly in the Al matrix [35], the HPT processing synthesized the intermetallic-based MMNC at the disk edge of the Al-Mg system.

After 10 HPT turns, carefully prepared disk edge was examined by XRD and the profile is shown in Figure 2c [34]. The result was examined by the MAUD software [36] so that the analysis quantified to give 73.4 ± 2.3% of Al, 4.9 ± 0.7% of Mg and 21.7 ± 1.5% of the intermetallic compound γ-Al_12_Mg_17_. It should be noted that the XRD analysis was not able to detect the presence of β-Al_3_Mg_2_ due to the small content that is smaller than the detectable limit. Moreover, although the disk edge was carefully prepared, the Mg-rich phase close to the central region was detected, whereas there is no presence of Mg-rich phase at the edge region in the Al-Mg system after HPT for 10 turns. The changes in lattice parameter of Al were calculated through the analysis to provide an estimate of the Mg solubility in the Al solid solution, and it is revealed that the Al matrix in the disk edge includes the supersaturate amount of Mg. Thus, the results showed that the disk edge after 10 turns involves two different types of intermetallic compounds forming a new type of MMNC.

The TEM micrographs taken at the peripheral region after HPT for 20 turns are shown in Figure 3a,b, where there is a mixture of microstructures with a nanolayered structure and an equiaxed grain structure, respectively. In practice, the nanolayered microstructure has an average thickness of ~20 nm and these layers contain numerous dislocations vertically. The equiaxed grains showed an average grain size of ~60 nm. By contrast, a PDA treatment demonstrated an apparent microstructural recovery, so that it is apparent from Figure 3c that the Al-Mg system contained a homogeneous equiaxed microstructure with an average grain size of ~380 nm [37].

The X-ray profiles are shown in Figure 3d,e for the carefully prepared Al-Mg disk edges after 20 turns by HPT and after HPT followed by PDA, respectively, where additional compositional analysis based on the X-ray profile through MOUD was displayed as a table in each plot [37]. It should be reminded once more that these disk edges after HPT for 20 turns do not include any Mg-rich phase, whereas the inevitable concentrations of the Mg-rich phase existed close to the mid-radius of the processed samples.

The disk edge immediately after HPT for 20 turns showed that there is evidence of γ-Al_12_Mg_17_ in the Al matrix, whereas, after PDA, there is an Al-7% Mg solid solution phase with two different intermetallic compounds of β-Al_3_Mg_2_ and γ-Al_12_Mg_17_. Thus, these results suggest that processing by HPT for 20 turns and additional PDA produced two different types of deformation-induced MMNCs containing intermetallic compounds at the disk edges of the Al-Mg system, and these MMNCs are different from those observed after five and 10 turns as shown in Figure 2. Moreover, the experimental results anticipate that the formation of these intermetallic compounds provides an excellent potential for reinforcing the Al matrix by improving the hardness and strength at the disk edges of the Al-Mg system.

### 2.2. Hardness Development in the Al-Mg Hybrid System

**Figure 4:**
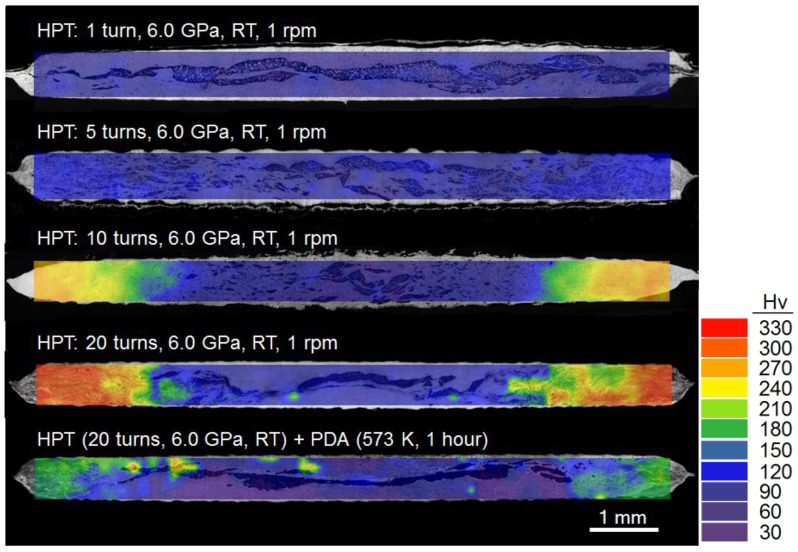
Color-coded hardness contour maps taken at the vertical cross-sections along the disk diameters after HPT for 1, 5, 10 and 20 turns [34] and after HPT for 20 turns followed by PDA.

The distributions of Vickers microhardness were examined over the vertical cross-sections of the processed Al-Mg disks as were shown in Figure 1, and the data set was visualized by constructing color-coded hardness contour maps that are then overlapped with the micrographs. These hardness maps are shown in Figure 4 for the disks after HPT for, from the top, 1, 5, 10 and 20 turns and after HPT followed by PDA, respectively. The hardness values are indicated in the color key on the right. For reference, the Al-1050 alloy and the ZK60 alloy show a saturation hardness of Hv ≈ 65 [38] and ~110 [39] across the disk diameters after HPT for five turns providing sufficient torsional straining.

The overall cross-section of the Al-Mg system after HPT for one turn shows an average microhardness value of ~70, which is similar to the saturated hardness value of ~65 for the base material of the Al alloy processed by HPT. This hardness value remains constant at the centers at *r* < 2.5–3.0 mm of the Al-Mg disks up to 20 turns. On the contrary, processing by HPT for five turns tends to introduce high hardness with a maximum of Hv ≈ 130 at the peripheral region where the fine Mg phase is homogeneously distributed within the Al matrix. Moreover, there is a significant increase in hardness after 10 turns where a maximum hardness of ~270 was recorded at the peripheral region at *r* > 3.0 mm. Moreover, exceptionally high hardness of Hv ≈ 330 was observed at *r* > 3.0 mm, where then the hardness shows the transition to ~150–240 at *r* ≈ 2.5–3.0 mm followed by Hv ≈ 60–70 at the central region at *r* < 2.5 mm. After PDA, the HPT-processed Al-Mg disk for 20 turns showed a slight reduction in hardness to Hv ≈ 30 in the central region at *r* ≤ 3.0 mm and to Hv ≈ 220 at the peripheral region at *r* ≈ 4.0–5.0 mm.

These high values of hardness at the Al-Mg disk peripheries are much higher than the highest achievable hardness in the base alloys of Al and Mg after HPT, and it is anticipated by the concurrent occurrences of grain refinement, solid solution hardening and precipitation hardening by the intermetallic compounds. It should be emphasized that the rapid diffusivity of Mg atoms into the Al matrix is a key process for the diffusion bonding of Al and Mg and for the formation of intermetallic compounds through HPT [35]. Limited recent studies demonstrated experimental evidence for enhanced atomic diffusion in bulk nanostructured materials processed by equal-channel angular pressing (ECAP) [40] and HPT [25,34]. The fast diffusivity in the SPD-processed materials may be attributed to the processing conditions including severe hydraulic pressure and a limited temperature rise during processing [34] and torsional stress [25] during HPT processing and the high population of lattice defects produced in the nanostructure [40]. A recent review describes the significance of the fast atomic mobility during SPD by recognizing the drastic increase in the vacancy concentration within the processed materials [41].

## 3. Micro-Mechanical Properties of the Al-Mg Hybrid System 

**Figure 5:**
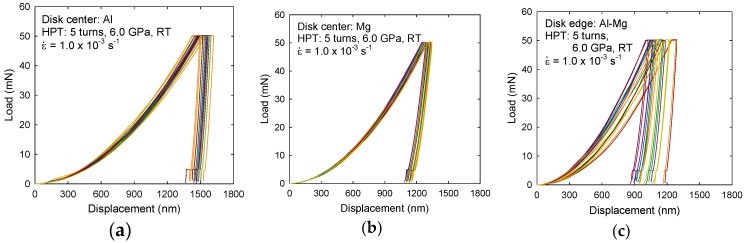
Representative load-displacement curves for (**a**) Al and (**b**) Mg phases at the disk center and for (**c**) the Al-Mg system forming an MMNC at the disk edge when testing at 1.0 × 10^−3^ s^−1^ for the sample after five turns by HPT [42].

The micro-mechanical response was examined using a nanoindentation technique for the Al-Mg system processed by HPT and after HPT with subsequent PDA. Figure 5 shows the representative curves of load, P, versus displacement, h, measured at an equivalent strain rate of 1.0 × 10^−3^ s^−1^ at the specific phases of (a) Al and (b) Mg at the disk center and (c) a mixture of Al and Mg phases and an intermetallic compound of β-Al_3_Mg_2_ at the disk edge after HPT for five turns [42]. It should be noted that the discontinuity of each curve at the final stage of unloading is due to thermal expansion, and these effects can be omitted from the analysis.

A series of indentation curves for the Al and Mg layers shows less broadening between the fifteen separate measurements as seen in Figure 5a,b, respectively. Therefore, it is demonstrated that there is a reasonably constant mechanical response within the phases in the multi-layered microstructure at the disk center. It is also apparent from these plots that higher ductility is shown in the Al phase with larger displacements under a fixed load in comparison with the Mg phase in the central region after HPT for five turns.

By contrast, Figure 5c shows a wide deviation in micro-mechanical response for all fifteen measurements, thereby indicating the plastic instability at the disk edge forming an MMNC in the Al-Mg system after five turns. It should be noted that, although the instability is demonstrated, all independent measurements showed smaller displacements than the separate Al and Mg phases without any intermetallic compound at the disk center as shown in Figure 5a,b. Thereby, it demonstrates higher hardness at the disk edge reinforced by an intermetallic compound and it is consistent with the hardness results demonstrated in Figure 4. Moreover, this plastic instability was observed at all indentation rates applied in the present measurements, although the slower strain rate shows a lower tendency.

**Figure 6:**
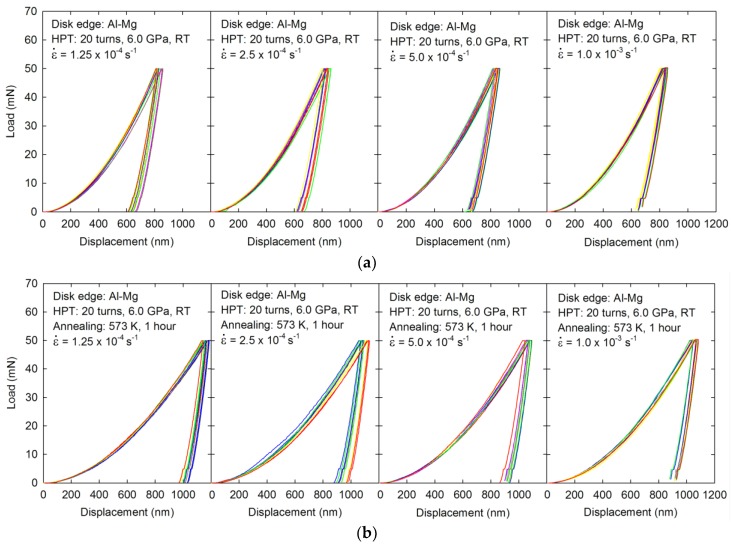
Representative load-displacement curves for the Al-Mg disk edges after (**a**) HPT for 20 turns and (**b**) HPT and PDA when measuring at four strain rates at 1.25 × 10^−4^ –1.0 × 10^−3^ s^−1^.

Figure 6 shows representative load-displacement curves for the Al-Mg disk edges after (a) HPT for 20 turns and (b) HPT followed by PDA when measuring at four equivalent strain rates at 1.25 × 10^−4^–1.0 × 10^−3^ s^−1^. The disk edge consists of an MMNC after 20 HPT turns showed all separate P-h curves at all four strain rates placed in reasonably consistent locations as shown in Figure 6a, thereby indicating no strain rate dependency of plasticity in the strain rate range. By contrast, a different type of MMNC processed by HPT followed by PDA revealed an apparent positive strain rate dependency where there are increasing displacements at slower strain rates of nanoindentation, as shown in Figure 6b. It is also apparent by comparing the load-displacement curves between the two samples that the MMNC immediately after HPT shows much lower displacements than the MMNC after HPT and PDA at all strain rates, thereby demonstrating the high hardness of the MMNC in the Al-Mg disk edge immediately after HPT for 20 turns. This result is fully consistent with the Vickers hardness measurements as shown in Figure 4.

It should be noted that no plastic instability was observed in both MMNCs at the disk edges after HPT for 20 turns and after PDA, which is in contrast with an MMNC in the Al-Mg system after HPT for five turns, as shown in Figure 5c. This apparent dichotomy is probably due to the higher volume fractions and the homogeneous distributions of the hard and fine phases of two intermetallic compounds within the microstructure after higher numbers of HPT turns.

Additional examinations were conducted by nanoindentation to demonstrate the hardness variations along the disk thicknesses at the central regions of the Al-Mg system after HPT for 20 turns and additional PDA. Specifically, the average values of nanoindentation hardness, H, were determined from three separate indentations recorded at uniformly separated points by a distance of 150 µm at the same distance from the mid-section in the disk height.

**Figure 7:**
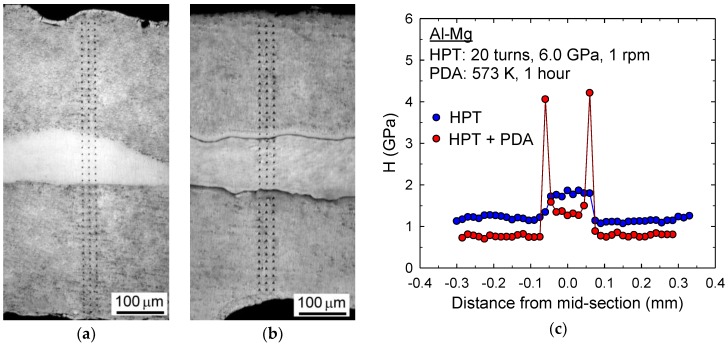
Optical micrographs showing the Al-Mg multi-layers at the disk centers with indentations for the samples after (**a**) HPT for 20 turns and (**b**) HPT and PDA and (**c**) variations of the nanoindentation hardness values for these two sample conditions of the Al-Mg system.

The optical micrographs showing the multi-layers at the disk centers with indentation marks are shown in Figure 7a,b for the Al-Mg disks after HPT for two turns and after HPT followed by PDA, respectively. It is apparent that the diffusion bonding of the Al and Mg phases were satisfactory so that there was no crack initiation by the severe stress introduced by the nanoindentation process at the interfaces in the multi-layers after 20 HPT turns. On the contrary, although there is consistent strong bonding at the interfaces, there are visible layers with an average thickness of <30 µm at the Al-Mg interfaces as shown in Figure 7b.

The variations of the measured indentation hardness are demonstrated in Figure 7c. The hardness immediately after HPT for 20 turns showed the apparent hardness changes at the Al-Mg interface boundaries. In practice, the hardness at the mid-section of the Al-Mg disk showed the higher hardness measured from the Mg phase, which is directly reduced with distance from the mid-section due to the phase change to Al. However, at the disk center after HPT and PDA, there is an exceptionally high hardness of over 4.0 MPa at the interphase boundaries, and it was determined as the β-Al_3_Mg_2_ by the chemical analysis.

Thus, although the hardness at the Al and Mg phases are reduced due to the microstructural recovery by PDA, it is concluded that the additional PDA process introduces further development in the diffusion-bonded interfaces in the Al-Mg system and demonstrating an excellent potential for constructing a unique microstructural formation throughout the disk diameter in such metal systems produced by HPT.

## 4. Discussion

### 4.1. Improvement in Micro-Mechanical Response by PDA

The deformation characteristics at RT were evaluated by calculating the strain rate sensitivity, *m*, from the data set of nanoindentation testing shown in Figure 5 and Figure 6 for the disk edges in the Al-Mg system after HPT for five and 20 turns, respectively. The value of *m* was determined at a given strain, ε, and absolute temperature, *T*, by considering Tabor’s empirical prediction showing that the flow stress is equivalent to H/3 for fully plastic deformation at a constant strain rate $\dot{\varepsilon }$ [43], where H is the nanoindentation hardness estimated according to the Oliver–Pharr method [44]:

(1) $$m={\left(\frac{\partial \ln\sigma_{f}}{\partial \ln\dot{\varepsilon }}\right)}_{\varepsilon ,T}={\left(\frac{\partial \ln\left(H/3\right)}{\partial \ln\dot{\varepsilon }}\right)}_{\varepsilon ,T}$$

**Figure 8:**
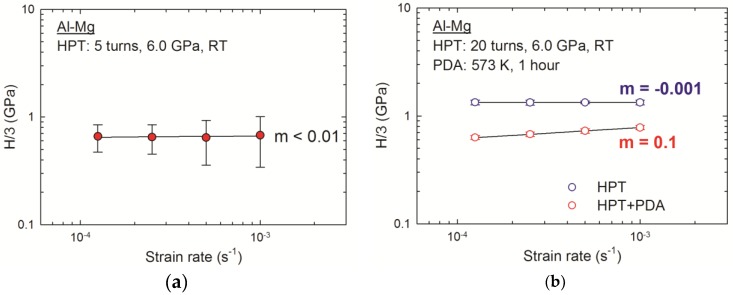
Variations of the strain rate sensitivity with increasing strain rate for the disk edges of the Al-Mg system after HPT for (**a**) five turns [42] and (**b**) 20 turns and after HPT and PDA [37].

Thus, the values of *m* were calculated from the slopes of the lines in a logarithmic plot of H/3 against $\dot{\varepsilon }$ as shown in Figure 8 for the disk edges of the Al-Mg system after HPT for (a) five turns [42] and (b) 20 turns and after HPT and PDA at 573 k for 1 h [37]. It should be noted that the error bar on each datum point represents the standard deviation of the numbers of measurements, whereas the error ranges are too small to recognize in Figure 8b.

The strain rate sensitivity was estimated as *m* ≈ 0.01 at the disk edge after HPT for five turns as shown in Figure 8a. Moreover, with wider error bars due to the plastic instability especially with increasing indentation strain rates, the estimations imply the possibility of a much smaller strain rate sensitivity in the Al-Mg system. However, the analysis estimated the *m* values of −0.001 and 0.1 for the MMNCs after HPT for 20 turns and HPT followed by PDA, respectively, as shown in Figure 8b. Thus, the strain rate sensitivity was reduced with increasing HPT turns, and, thereafter, the PDA treatment provided a significant enhancement in the *m* value of the MMNC in the Al-Mg system processed by HPT. A recent review tabulated the available data of the strain rate sensitivity, *m*, in a series of UFG metals processed by SPD [28].

There is a limited report of a negative strain rate sensitivity for an MMNC of a powder consolidated aluminum 6092/B_4_C when tested at strain rates of <1.0 s^−1^ [45]. The report suggested the occurrence of dynamic strain ageing (DSA) due to the presence of the fast diffusion of solute atoms interacting with mobile dislocations. Thus, at the present MMNC in the Al-Mg system immediately after HPT, decreasing plasticity demonstrated by the reduced strain rate sensitivity is reasonable because of the formation of dislocation junctions at the solute clusters, which is attributed to interaction of a significant number of dislocations created during HPT with the very rapid Mg solutes within Al matrix.

The improved *m* value of 0.1 for the PDA-treated MMNC in the Al-Mg system processed by HPT is even higher than the reported *m* values through nanoindentation testing of ~0.07 for a commercial purity Al after ECAP for 6–12 passes at RT [14,15,17,18,20] and after accumulative roll bonding (ARB) for eight cycles at RT [15] and ~0.035–0.050 for a ZK60 alloy after HPT for two turns at RT [21]. Thus, the MMNC after HPT followed by PDA demonstrates a significantly higher value of *m* compared with the initial materials when they are processed separately.

The present study demonstrated that a PDA treatment is feasible for enhancing the plasticity of the MMNCs in the Al-Mg system after HPT while maintaining reasonably high hardness as shown in Figure 4. In practice, this approach produces an ordering of the defect structures within the grain boundaries leading to an equilibrium state without any significant grain growth [46]. In addition, short-term annealing reduces the dislocation density in the grain interior of the UFG material after SPD so that the dislocation storage capability may increase and thus the strain hardening capability is enhanced. Thus, this leads to the possibility of high ductility in the SPD-processed material and there are several recent reports demonstrating the significance of PDA on mechanical properties at RT of HPT-processed materials [47,48,49,50].

### 4.2. Future Potential of a Nanoindentation Technique for UFG Metals

Recent developments in characterization techniques result in a better understanding of the enhancement in the mechanical properties of UFG materials processed by SPD. In particular, the novel technique of nanoindentation has become a common tool for the simultaneous measurement of a number of mechanical properties on the material surfaces at the submicron scale. [13]. Accordingly, there have been several studies to date demonstrating the use of the nanoindentation technique for examining the mechanical properties and parameters of SPD-processed metals [15], while numerous studies demonstrated the conventional procedures of mechanical testing in tension and compression using bulk UFG metals.

The attraction of a nanoindentation technique arises from the requirement of limited volumes, which is favorable for the SPD-processed materials where, with the current laboratory-scale studies on the SPD techniques, the tensile specimens machined from SPD-processed samples have often failed to meet the geometry defined by the American Society for Testing and Materials (ASTM) standard [51]. Moreover, several recent studies demonstrated a new scale of engineering materials processed by SPD techniques where the microstructure includes a gradient-type nanostructure according to grain size and composition [34,52]. This type of structural organization is defined as heterogeneous architecture materials [53] and is also demonstrated as a new type of structure in engineering materials leading to a significant potential for exhibiting excellent mechanical properties [54,55,56,57]. Thus, for understanding the local mechanical properties with microstructural gradations in length scales, the nanoindentation technique has been indispensable for measuring the specific mechanical response using a limited volume of material at arbitrarily selected local points.

Finally, it is reasonable to note that a recent review of nanoindentation describes the role, significance and feasibility of the nanoindentation technique [15]. Moreover, very recent reports describe current advances and capabilities of the novel nanoindentation techniques for measuring thermally-activated deformation mechanisms ranging from single crystalline to nanocrystalline metals through strain rate jump testing and long term creep testing [58] and for operating at high temperature in situ in a scanning electron microscope (SEM) [59].

## 5. Materials and Methods

**Figure 9:**
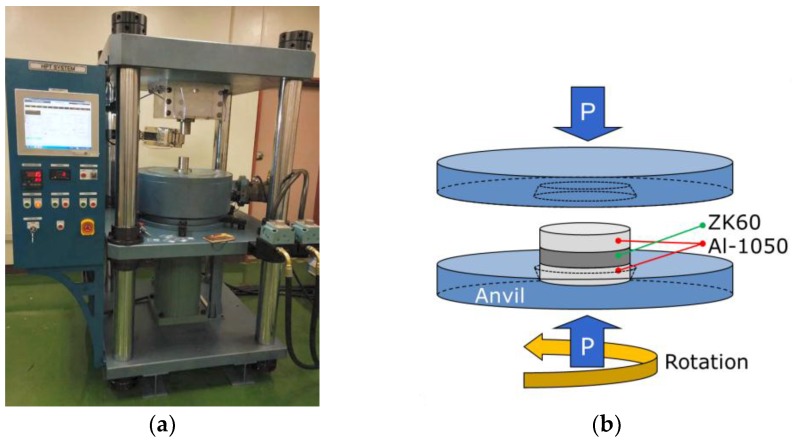
(**a**) Photograph of the HPT facility and (**b**) schematic illustration of the sample set-up for HPT processing [34,35].

A commercial purity Al-1050 alloy and a ZK60 magnesium alloy were used for the experiments. The extruded bars of the alloys having a diameter of 10 mm were cut into billets with lengths of ~65 mm and a set of disks was sliced from the billets and polished to achieve uniform thicknesses of ~0.83 mm. The direct bonding of the Al and Mg disks was performed through the application of the conventional HPT technique at RT following the general processing procedure described earlier [60] under a hydraulic pressure of 6.0 GPa for 1 to 20 turns at a rotational speed of 1 rpm. In practice, separate disks of the Al and Mg alloys were placed in the depression on the lower anvil on the order of Al/Mg/Al, where the Mg disk was positioned between the two Al disks but without using any glue or metal brushing treatment. Figure 9 shows (a) a picture of the HPT machine and (b) the stacked disks of Al and Mg between the conventional set-up of the HPT anvils [34,35]. In order to evaluate the effect of PDA, some of the HPT-processed disks after 20 turns were annealed at 573 K for 1 h.

Following processing, vertical cross-section from each processed disk was polished, chemically etched using Keller’s etchant and examined by optical microscopy (OM). Subsequently, the values of Vickers microhardness, Hv, were recorded over the vertical cross-sections of the disks after HPT and HPT followed by PDA using a Shimazu HMV-2 facility (Kyoto, Japan) with a load of 50 gf. These individual microhardness values were recorded following a rectilinear grid pattern with an incremental spacing of 0.2 mm.

An elemental analysis was conducted using energy-dispersive X-ray spectroscopy (EDS) in a field emission scanning electron microscope (FE-SEM), FEI Nova NanoSEM 450 (Hillsboro, OR). The detailed microstructure was investigated by FE-SEM, JEOL JSM-6700F (Tokyo, Japan), in the peripheral region near the outer edge of the disks on the vertical cross-section after preparing using a broad ion beam cross-section polisher, JEOL IB-09020CP, with 6 kV Ar ion beam and 30° swing angle of specimen stage to minimize beam striations on the strain-free polished surface. An X-ray diffraction (XRD) analysis was performed using a Rigaku UltimaIV XRD (Tokyo, Japan) on the slightly polished surface of each disk. The examination used a CuKα radiation with a scanning speed of 3° min^−1^ and a step interval of 0.01°. Microstructural cell parameters and phase percentages were quantified by means of the XRD data analysis software, Materials Analysis Using Diffraction (MAUD), which is based on a full pattern fitting procedure (Rietveld method). Additional microstructural analysis was conducted by transmission electron microscopy (TEM) using a spherical aberration (Cs) corrected JEOL JEOM-2100 F with 200 kV accelerating voltage for a specimen prepared by an in situ lift-out technique using OmniProbe 200 (Oxfordshire, U.K.) and Omni gas injection system (GIS) in a focused ion beam (FIB), JEOL JIB-4500.

The micro-mechanical response was examined at the disk centers and the MMNC at the disk edges in the Al-Mg system after HPT for 5 and 20 turns and after PDA at RT using a nanoindentation facility, Nanoindenter-XP (formerly MTS; now Keysight, Santa Rosa, CA, USA), with a three-sided pyramidal Berkovich indenter having a centerline-to-face angle of 65.3°. More than 15 indentations were conducted at each specific phase at the measured locations to provide statistically valid data. All measurements were conducted under a predetermined peak applied load of P_max_ = 50 mN at constant indentation strain rates of 0.0125, 0.025, 0.05 and 0.1 s^−1^, which are equivalent to general strain rates of 1.25 × 10^−4^, 2.5 × 10^−4^, 5.0 × 10^−4^ and 1.0 × 10^−3^ s^−1^ calculated through an empirical relationship [61,62].

## 6. Conclusions

There is a considerable potential for making use of HPT for the introduction of new alloy systems, especially for fabricating a wide range of hybrid materials. The nanoindentation technique provides a wide range of information including mechanical properties and the local microstructure. In addition to conventional tensile testing, this technique is promising for UFG materials processed by HPT, where the materials may have smaller overall dimensions and include gradient-type microstructures. Further investigations are needed to fully develop this approach.
